# EXPLORING AGEISM WITHIN THE FAMILY

**DOI:** 10.1093/geroni/igac059.2433

**Published:** 2022-12-20

**Authors:** Stacey Gordon, Ernest Gonzales

**Affiliations:** New York University, New York, New York, United States; New York University, New York, New York, United States

## Abstract

It is well documented that ageism negatively affects older people's opportunities for productive aging, and access to the workplace and healthcare. Ageism as it manifests within families, however, has not yet been well studied despite significant implications for the wellbeing of older adults and families. This study presents the problem of ageism in the family through the lens of larger social structural factors shaping meso- and micro-level behaviors. Negative implicit ageism and stereotype embodiment theory underscore the unconscious perpetuation of negative age stereotypes and attitudes in families. To illustrate this concept, we describe three evidence-based case examples involving interactions among family members observed by a geriatric care manager. The first describes how ageism expressed by an adult child undermines choice, opportunity, and power of the parent when an older adult enlists the help of her adult children in downsizing from the family home to a smaller apartment. The second explores ageism when adult children attempt to persuade their older father to leave his home, pointing out dangers living alone at his age. The third describes a relatively healthy newly widowed woman who is coerced by her adult children to move to an assisted living facility by threatening social and emotional abandonment. These examples demonstrate how macro and meso-level factors combine with the implicit ageism of both adult children and older adults converge, influencing pathways to create healthy, tolerable and toxic living conditions within the family.

